# Post-translational modifications as key regulators of TNF-induced necroptosis

**DOI:** 10.1038/cddis.2016.197

**Published:** 2016-07-07

**Authors:** X Liu, F Shi, Y Li, X Yu, S Peng, W Li, X Luo, Y Cao

**Affiliations:** 1 Cancer Research Institute, Xiangya School of Medicine, Central South University, Hunan, China; 2 Key Laboratory of Chinese Ministry of Education, Central South University, Hunan, China; 3 Key Laboratory of Carcinogenesis of Chinese Ministry of Public Health, Central South University, Hunan, China; 4 The Affiliated Cancer Hospital of Xiangya School of Medicine, Central South University, Hunan, China; 5 The Third Xiangya Hospital, Central South University, Hunan, China

## Abstract

Necroptosis is a novel form of programmed cell death that is independent of caspase activity. Different stimuli can trigger necroptosis. At present, the most informative studies about necroptosis derive from the tumor necrosis factor (TNF)-triggered system. The initiation of TNF-induced necroptosis requires the kinase activity of receptor-interacting protein 1 and 3 (RIP1 and RIP3). Evidence now reveals that the ability of RIP1 and RIP3 to modulate this key cellular event is tightly controlled by post-translational modifications, including ubiquitination, phosphorylation, caspase 8-mediated cleavage and GlcNAcylation. These regulatory events coordinately determine whether a cell will survive or die by apoptosis or necroptosis. In this review, we highlight recent advances in the study of post-translational modifications during TNF-induced necroptosis and discuss how these modifications regulate the complex and delicate control of programmed necrosis.

## Facts

The ability of receptor-interacting protein 1 and 3 (RIP1 and RIP3) to modulate necroptosis is tightly controlled by post-translational modifications, including ubiquitination, phosphorylation, caspase 8-mediated cleavage and N-acetylglucosaminylation (GlcNAcylation).
K63-polyubiquitinated RIP1 is retained at the plasma membrane and serves as a docking site to mediate NF-*κ*B activation, whereas K63-polyubiquitinated RIP3 supports the formation of the RIP1–RIP3 complex and subsequent induction of necroptosis.
RIP1 and RIP3 autophosphorylate themselves and also phosphorylate each other.
Caspase 8 cleaves RIP1 on Asp324 and RIP3 on Asp328, which abolishes the activity of RIP1 and RIP3 and thus inhibits necroptosis.

## Open Questions

Which enzymes mediate the ubiquitination of RIP3?
Is Ser161 phosphorylation critical for the pro-necrotic function of RIP1?
Knockdown of phosphoglycerate mutase family member 5 (Pgam5) or dynamin-related protein 1 (Drp1) was able to inhibit tumor necrosis factor (TNF)-induced necroptosis in HeLa cells, whereas it had no effect on necroptosis in MEF, L929, SVEC and NIH-3T3 cells. Is Pgam5–Drp1 axis cell-type-specific?
Does acetylation mediate necroptosis?

Programmed necrosis or necroptosis is an alternative form of programmed cell death that is triggered when apoptosis is inhibited.^1, 2^ Similar to apoptotic cell death, programmed necrosis can also be initiated by death ligands (such as TNF and Fas), interferons, Toll-like receptor (TLR) ligands and microbial infection.^3, 4, 5, 6, 7, 8, 9^ In the aspect of host defense against infection, necroptosis could be considered as a back-up mechanism for host cells to contain pathogen replication and spread when the apoptotic pathway is compromised.^10^ Unlike apoptosis, which is a caspase-dependent process characterized by chromatin condensation and nuclear fragmentation, necroptosis is a caspase-independent pathway characterized by morphological features resembling necrosis.^1^ This death pathway is involved in many pathological processes, such as chronic inflammation,^11, 12^ kidney ischemia/reperfusion injury,^13^ neurodegenerative disease,^14^ retina detachment^15^ and atherosclerosis.^16^

The implication of RIP1 in necroptosis was first discovered in 2000 and was definitively confirmed by the identification of necrostatin-1 in 2008, which specifically targets RIP1 kinase and thus inhibits necroptotic cell death.^4, 17^ Subsequently, RIP3 was reported to physically and functionally interact with RIP1 to generate a super molecular complex during necroptosis.^7, 18^ To distinguish this complex from other complexes that induce apoptosis or nuclear factor-*κ*B (NF-*κ*B) activation, the RIP1–RIP3 complex has been termed the 'necrosome'.^19^ Interestingly, RIP1 and RIP3 belong to the same kinase family, and each of them has an N-terminal kinase domain. Under the stimulation of TNF/zVAD, RIP1 and RIP3 form a heterodimeric scaffold through their respective RIP homotypic interaction motif domains.^20^ The necrosome recruits and activates downstream substrates such as mixed lineage kinase domain like protein (MLKL) to execute necroptosis.^21^ Therefore, a better understanding of the regulatory mechanism of necrosome will be helpful to reveal the process of necroptosis.

**Figure 1:**
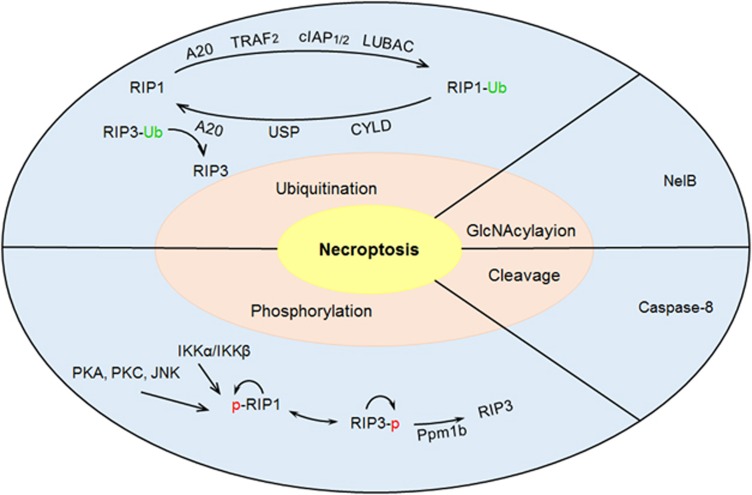
Post-translational modifications regulate necroptosis. The process of necroptosis is tightly controlled by post-translational modifications, including ubiquitination, phosphorylation, caspase 8-mediated cleavage and GlcNAcylation

Post-translational modifications have crucial roles in regulating protein function, and thereby control several fundamental aspects of eukaryotic biology, including signal transduction, protein trafficking, cell–cell interactions, cell proliferation and differentiation. Indeed, emerging evidence indicates that necrosome assembly and activation is tightly controlled by post-translational modifications. The aim of this review is to give an overview on the role of post-translational modifications in the process of necroptosis, especially focusing on protein ubiquitination, phosphorylation, caspase 8-mediated proteolytic cleavage and GlcNAcylation (Figure 1).

## The Diverse Role of Protein Ubiquitination in Necroptosis

Ubiquitylation is among the most widely used protein modifications involved in regulating cellular signaling and homeostasis. As an ubiquitin contains seven lysines (Lys6, Lys11, Lys27, Lys29, Lys33, Lys48 and Lys63), there are seven possible types of homotypic linkage as well as linear ubiquitin linkage. The ubiquitin can be inducibly and reversibly attached to a range of proteins and then regulate a multitude of cellular functions.^22^ For example, K48-linked polyubiquitination mainly targets protein for proteasomal degradation, whereas K63-linked polyubiquitination typically regulates protein function, subcellular localization and protein–protein interactions.

### The regulation of RIP1 ubiquitination

In the TNF*α*-induced NF-*κ*B signaling pathway, RIP1 is a dual-functional signaling molecule that is capable of either pro-survival or pro-death, depending on the state of K63-linked polyubiquitination.^23^ Upon TNF stimulation, TNF receptor 1 (TNFR1) recruits multiple proteins to form a transient molecular complex, named complex I. This complex consists of TRADD (TNF receptor-associated death domain), TRAF2 (TNF receptor-associated protein 2), cIAP (cellular inhibitor of apoptosis protein) and RIP1. In complex I, RIP1 is modified by multiple forms of ubiquitination, including linear, K63 and K11-linked polyubiquitinations. K63-linked polyubiquitination of RIP1 functions as a docking site for the TAK1–TAB2–TAB3 complex and for NEMO, both of which are crucial for the activation of NF-*κ*B and provide survival signals. Deficient complex I activity, such as deubiquitination of RIP1, inhibits NF-*κ*B activation and leads to the formation of one of the two alternative cytosolic complexes, complex IIa or complex IIb.^24^ Complex IIa includes Fas-associated death domain (FADD), caspase 8 and RIP1, which activates downstream caspases and subsequent apoptosis. When the activation of caspase 8 is inhibited, RIP1 and RIP3 interact with each other to form complex IIb, which can induce necroptosis. The conversion from complex I to complex II is an important regulatory step. To date, multiple deubiquitinating enzymes (DUBs), E3 ubiquitin ligases and linear ubiquitin ligases, have been identified to modulate the deubiquitination or ubiquitination of RIP1 in the TNF signaling pathway.

It is known to all that both the deubiquitination and ubiquitination are tightly mediated by DUBs and ubiquitination enzymes. Interestingly, A20 is an unusual protein that has dual ubiquitinating and deubiquitinating activity. The C-terminal zinc-finger (Znf) domain of A20 acts as an E3 ligase by polyubiquitinating RIP1 with K48-linked ubiquitin chains, thereby targeting RIP1 for proteasomal degradation.^25^ It is worth noting that the Znf domain of A20 activates not only K48-linked but also K63-linked polyubiquitin chains. In human glioblastoma, the A20 Znf domain mediates K63-linked polyubiquitination of RIP1 and thus inhibits cell death.^26^ The N-terminal ovarian tumor domain of A20 acts as a DUB by removing K63-linked polyubiquitin chains from RIP1, which consequently downregulates TNF*α*-induced NF-*κ*B signaling.^25^ As discussed above, the role of A20 is much more complicated than that of an individual DUB or E3 ligase for its two opposite-function domains. Therefore, exploring its role in cell survival and death will shed light on the precise biological function of A20.

The ubiquitin-specific proteases (USPs) become DUBs by cleaving ubiquitin adducts from specific protein substrates, thereby regulating protein activation or protecting the substrate from proteasome-mediated degradation. The USP family members USP2a, USP4 and USP21 are believed to inhibit RIP1-mediated NF-*κ*B activation and promote TNF*α*-induced cell death by removing K63-linked polyubiquitination chains from RIP1 in the TNFR1 complex.^27, 28, 29^ Another DUB, cylindromatosis (CYLD), may also function as a switch in regulating the activation of necroptosis over NF-*κ*B activation and cell survival in three aspects. First, CYLD inhibits NF-*κ*B signaling by deubiquitinating NF-*κ*B-positive regulators, such as TAK1 (TGF-*β*-activated Kinase 1), TRAF2 and NEMO/IKK*γ*.^30, 31^ Second, caspase 8-mediated cleavage of CYLD generates a survival signal, whereas the mutation of caspase 8-mediated cleavage site on CYLD switches cell survival to necrotic cell death in response to TNF*α*.^32^ Last but not least, CYLD interacts with and deubiquitinates RIP1.^33^ However, it is still controversial whether CYLD affects the ubiquitination of RIP1 in complex I or in the necrosome.^34, 35^ Given that the above DUBs can remove ubiquitin chains from RIP1, how is RIP1 ubiquitinated?

It is worth noting that cIAP1/2 are RING domain-containing E3 ubiquitin ligases that have exceptional capacity to promote K63-, K48- and K11-linked polyubiquitination of RIP1. At first, it was found that cells deficient in cIAP1/2 are defective in RIP1 ubiquitination and NF-*κ*B activation after TNF*α* stimulation.^36, 37, 38^ These researches revealed that cIAP1/2 have a critical role in protecting against TNF*α*-induced cell death by promoting K63- and K48-linked polyubiquitination of RIP1. cIAP1/2 also mediate the assembly of K11-linked ubiquitin chains on RIP1.^39^ Unlike K63 and K48 linkages, K11-linked ubiquitin chains have been less studied and are considered to serve as a degradation signal. However, Dynek *et al.*^39^ demonstrated for the first time that K11 ubiquitin linkage may take a part in TNF signaling, as the adaptor protein NEMO can be recruited to K11 ubiquitin chains of RIP1. As discussed above, cIAP1/2 act as E3 ligases to promote K63, K48, as well as K11-linked polyubiquitination of RIP1 in the TNFR1 complex, which facilitate cell survival and protect against cell death. Nevertheless, the role of another E3 ubiquitin ligase TRAF2 within the TNFR signaling complex is controversial. On the one hand, genetic evidence indicates that TRAF2 is responsible for the K63-linked ubiquitin chains of RIP1, as RIP1 polyubiquitination is not observed in response to TNF*α* in TRAF2 KO cells.^40, 41^ The RING domain of TRAF2 with E3 activity is responsible for polyubiquitination of RIP1 and inhibition of cell death induced by TNF*α*.^40^ On the other hand, it is believed that TRAF2 is critical for recruitment of cIAP1/2,^42^ E3 ligases that are indeed essential for RIP1 ubiqutination within the complex. The recent finding that TRAF2 suppresses TNF*α*-induced necroptosis through competing with RIP3 for MLKL binding provides a crucial element in our understanding of the biological function of TRAF2.^43^

The most recently described linear (head-to-tail) ubiquitin chain is another type of non-degradative signaling modification that functions as an important cellular regulator.^44^ This kind of ubiquitin chain is assembled by a specific ligase complex called linear ubiquitin chain assembly complex (LUBAC), which contains two RING-type E3 ligases, heme-oxidized IRP2 ubiquitin ligase 1 (HOIL1) and HOIL1-interacting protein (HOIP), and an adaptor protein Sharpin. LUBAC catalyses the formation of a peptide bond between the N-terminal methionine of one ubiquitin molecule and the C-terminal glycine of the next, which is the so-called head-to-tail ubiquitination.^45, 46^ The importance of linear ubiquitylation to necroptosis was brought to light by knockdown of HOIL1/HOIP, and mutation of Sharpin. It was worth noting that the repression of LUBAC component HOIL1 or HOIP enhanced TNF-induced necroptosis.^47^ Similarly, the mutation of Sharpin led to TNF-dependent inflammatory syndrome, characterized by dermatitis, liver inflammation, splenomegaly and loss of Peyer's patches.^48^ Although loss of a single allele of Casp8 was strikingly effective in delaying the onset of dermatitis, it did not have an impact on other aspects of phenotype. Unlike Casp8 heterozygosity, RIP3 and MLKL deletion resulted in reduced splenomegaly and completely rescued liver inflammation.^49^ Taken together, these results indicate that the absence of Sharpin leads to aberrant apoptosis in certain tissues and necroptosis in others. Given that a direct regulation of RIP1 by LUBAC is not yet clear, it will be necessary to delve further into the mechanism of LUBAC-mediated control of RIP1.

### The regulation of RIP3 ubiquitination

**Figure 2:**
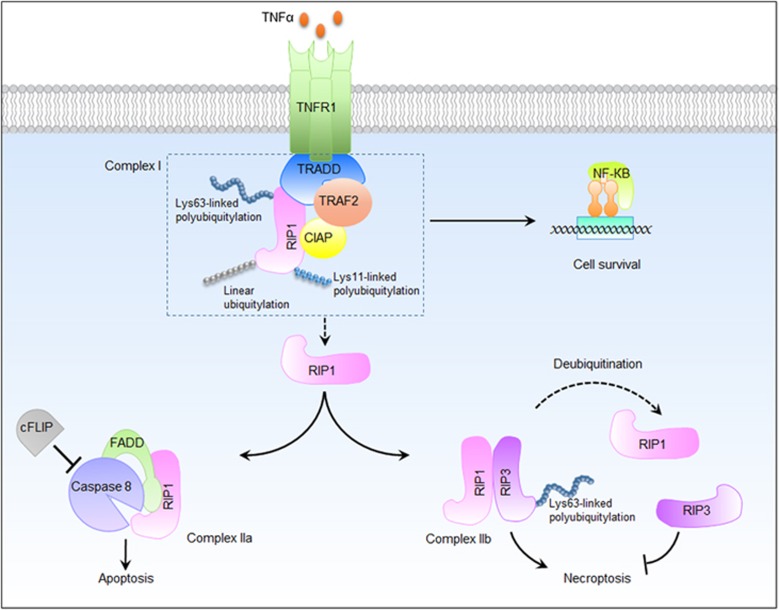
The diverse role of RIP1 and RIP3 ubiquitination in TNF*α*-induced necroptosis signaling. Upon stimulation with TNF*α*, TNFR1 recruits TRADD, which provides a scaffold for the assembly of complex I at the plasma membrane by binding with RIP1, TRAF2 and cIAP. In complex I, RIP1 is polyubiquitylated by Lys63-linked and Lys11-linked ubiquitin chains as well as linear ubiquitin chains, and further mediates the activation of NF-*κ*B. Deubiquitination of RIP1 or the inhibition of cIAP promotes the conversion of complex I to complex II. Complex IIa contains RIP1, FADD and caspase 8. Normally, caspase 8 cleaves RIP1 and RIP3, and triggers apoptosis. In the presence of cellular FLICE-like inhibitory protein, caspase 8 is unable to initiate apoptosis but maintains sufficient basal protease activity to cleave RIP1 and RIP3. Once caspase 8 is blocked by pharmacological or genetic intervention, RIP1 and RIP3 interact with each other to form complex IIb, which contributes to the downstream events of necroptosis. Lys63-linked polyubiquitination of RIP3 is required for the formation of this complex. The deubiquitination of RIP3 suppresses RIP1–RIP3 interaction and inhibits RIP3-dependent necroptotic cell death

Although the ubiquitination pattern of RIP1 has been shown to provide a unique ‘ubiquitin code', which determines whether a cell will survive or die, the importance of RIP3 ubiquitination was not known until now. The latest research indicates that K63-linked polyubiquitination of RIP3 at Lys5 is required for the formation of the RIP1–RIP3 complex and induction of RIP3-mediated necroptosis. The DUB A20 restricted the ubiquitination of RIP3 and RIP1–RIP3 interaction through its deubiquitinating motif. Therefore, A20 inhibits RIP3-dependent necroptotic cell death.^50^ This finding sheds new light on the importance of RIP3 ubiquitination in the process of necroptosis (Figure 2). However, there are still numerous fundamental problems remaining to be investigated. (1) Which enzymes mediate the ubiquitination of RIP3? (2) Besides A20, is there any other DUB that could block RIP3 polyubiquitination? (3) What is the role of RIP3 ubiquitination in cell survival and death? Therefore, there is still a long way to go before we have a clear and unequivocal understanding of RIP3 ubiquitination.

## Protein Phosphorylation Regulates Necrosome Assembly and Activity

The role of phosphorylation as a significant cellular signal has been established in many biological processes, much like ubiquitination. Introduction of a phosphoryl group will add negative charge to the substrate protein and increase the size of the residue sharply, therefore altering the global protein conformation. Most importantly, phosphorylation events can modulate enzymatic activity of the phosphoprotein, affect protein stability, influence cellular localization and confer the change of intermolecular interactions in protein complexes.^51^ Protein phosphorylation on Ser, Thr, Tyr, His and Asp has been identified to take the key regulatory roles in numerous cellular processes, such as cell growth, cell differentiation, cell cycle and cell migration.^52, 53, 54^ In recent studies, it has been found that the reversible protein phosphorylation also has a critical role in the process of necroptosis. It is particularly important that the programmed necrosis induced by TNF*α* is dependent on the kinase activity of RIP1 and RIP3. As a result, these kinases are potential targets for discovering necroptosis inhibitors.

### The regulation of RIP1 phosphorylation

As a serine–threonine protein kinase, RIP1 can be phosphorylated either by itself (auto) or by other kinase(s). It is noteworthy that RIP1 phosphorylation was completely abrogated in RIP3-knockout cells.^7^ Although the level of RIP3-mediated RIP1 phosphorylation was low, RIP3 was still considered to act as an upstream regulator of necrosis-specific RIP1 phosphorylation. The phosphorylation analysis by using immobilized metal affinity chromatography and mass spectrometry identified a number of phosphorylated residues on RIP1.^17^ Among them, Ser14/15, Ser20, Ser161 and Ser166 represent RIP1 autophosphorylation sites, whereas Ser6, Ser25, Ser303, Ser320, Ser330/331 and Ser333 are likely to be phosphorylated by other kinase(s). However, the functional significance of these phosphorylation sites has not been well demonstrated, yet except for Ser161. The homology modeling of RIP1 on B-RAF suggested that Ser161 phosphorylation may be involved in the regulation of RIP1 kinase activity. Following this, some researchers observed that S161A mutation significantly decreased RIP1 kinase activity and attenuated 40% necroptosis.^17^ However, other studies found that S161A only reduced RIP1 kinase activity by ~20%, which was not sufficient to reverse programmed necrosis.^55^ Considering these points, it is not easy to draw a conclusion whether Ser161 phosphorylation is critical for the pro-necrotic function of RIP1. However, it is certain that Ser161 contributes to necrostatin-1 (Nec-1)-mediated inhibition of RIP1 kinase activity.^17^

Recently, the inhibitory phosphorylation sites of RIP1 have been reported. At first, researchers identified a novel phosphorylation site on RIP1, Ser89, which can dampen the pro-necrotic function of RIP1.^55^ As S89A mutation resulted in hyperactive RIP1 kinase activity and increased TNF*α*-induced programmed necrosis, it is suggested that Ser89 phosphorylation inhibits RIP1 kinase activity and suppresses RIP1-dependent programmed necrosis. Protein kinase (PK) A, PKC or c-Jun N-terminal kinase may act as the kinase that phosphorylates RIP1 at this site. After that, another inhibitory phosphorylation site of RIP1 is reported to be mediated by the inhibitor of NF-*κ*B kinase (IKK) complex.^56^ It is demonstrated that IKK*α*/IKK*β* directly phosphorylates RIP1 in complex I, which either represses RIP1 kinase activity or interferes with RIP1's ability to bind complex IIb components, thereby protecting cells from RIP1 kinase-dependent death. The potential candidates for IKK*α*/IKK*β*-mediated phosphorylation of RIP1 are S25, S166, S296, S331 and S416. The findings from the above research suggest that RIP1 phosphorylation has a dual role in the regulation of programmed necrosis.

### The regulation of RIP3 phosphorylation

**Figure 3:**
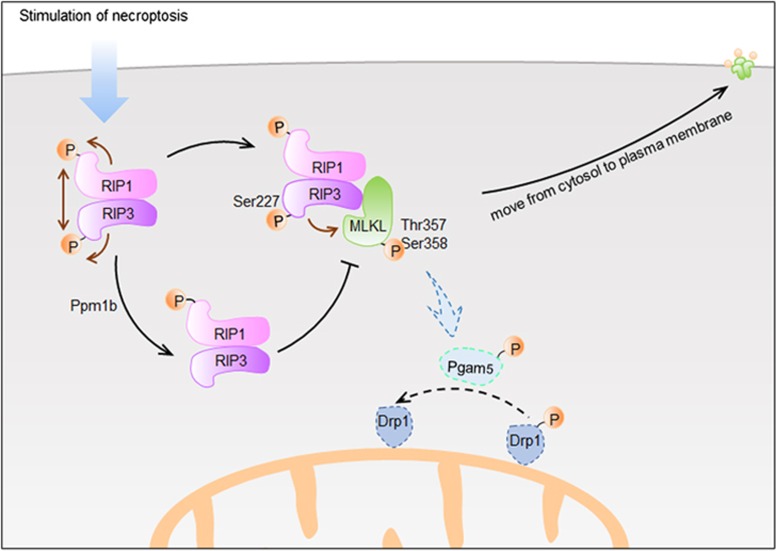
The phosphorylation events during necroptosis. Upon the stimulation of necroptosis, RIP1 and RIP3 form a stable complex, termed the 'necrosome'. In this complex, RIP1 and RIP3 autophosphorylate themselves and also phosphorylate each other. Phosphorylation of human RIP3 on Ser227 (Thr231/Ser232 for mouse RIP3) is particularly important for recruitment of MLKL. Ppm1b negatively regulates necroptosis through dephosphorylating RIP3 and thus prevents the recruitment of MLKL to necrosome. After binding with RIP3, MLKL is phosphorylated by RIP3 at Thr357 and Ser358. Upon phosphorylation, MLKL oligomerizes and translocates from the cytosol to plasma membrane, which leads to membrane rupture. In some cases (for example, in HeLa cells), RIP3 mediates phosphorylation of mitochondrial phosphatase Pgam5, which dephosphorylates the mitochondrial fission regulator Drp1 and leads to extensive mitochondrial fission

The phosphorylation of RIP3 is also a crucial event during programmed necrosis. Interestingly, RIP1 kinase activity is required in this process as RIP3 phosphorylation is inhibited by RIP1 kinase inhibitor Nec-1.^7^ McQuade *et al.*^55^ revealed that the phosphorylation on Ser204 in mouse RIP3, which regulates the pro-necrotic function, is facilitated by RIP1. Furthermore, RIP3 appears to be autophosphorylated at Ser199 and Ser227 in human cells, and the Ser227 phosphorylation is required for human RIP3 to interact with human MLKL in the necrosome.^21, 57^ Similarly, the Thr231 and Ser232 autophosphorylation on mouse RIP3 is essential for the recruitment and activation of mouse MLKL.^58^ Although there are many other phosphorylation sites on mouse RIP3 (Ser2, Ser165, Thr257, Ser304, Ser326, Thr338, Ser353, Ser369, Ser380 and Thr392), they seem to be unnecessary for mouse RIP3 to mediate necroptosis.^58^ Recently, protein phosphatase 1b (Ppm1b) has been identified first as a nonspecific RIP3 phosphatase that negatively regulates necroptosis through dephosphorylating RIP3 and suppressing MLKL recruitment to the necrosome.^59^ According to what has been discussed above, RIP1 phosphorylation could promote or inhibit its kinase activity, which is required for necrosome formation, whereas RIP3 phosphorylation is essential for RIP3–MLKL interaction (Figure 3).

### The downstream (de)phosphorylation events

MLKL is a key necroptotic effector downstream of RIP3 in TNF*α*-induced necroptosis, which consists of an N-terminal coiled-coil domain and a C-terminal kinase-like domain. To date, controversial data have been reported on whether MLKL itself has kinase activity. Some studies found that MLKL is catalytically inactive,^21, 60^ whereas others considered MLKL as an atypical kinase with weak kinase activity.^61^ MLKL binds to RIP3 through its kinase-like domain, which is phosphorylated by RIP3 at Thr357 and Ser358 in human cells.^21^ Different from human MLKL, Ser345, Ser347 and Thr349 are mouse MLKL phosphorylation site mediated by RIP3.^62^ The latest study showed that the phosphorylation of Ser345 was a key event in the activation of MLKL by RIP3 instead of the other two.^63^ The phosphorylation sites mutant of MLKL retained the formation of the RIP3–MLKL complex.^64^ However, it was unable to reconstitute necroptosis.^21^ These results suggest that the binding between RIP3 and MLKL is necessary but not sufficient for necrosis signaling. Upon phosphorylation, MLKL forms an oligomer that moves from the cytosol to plasma and intracellular membranes, thereby disrupting membrane integrity and resulting in necrotic death.^65, 66, 67^ As phosphorylated MLKL is a marker of necroptosis, the antibodies that detect phospho-MLKL are considered to be one of the effective methods for detecting necroptosis occurred *in vitro* and *in vivo*.

Pgam5, a mitochondrial Ser/Thr protein phosphatase, has been identified to be another substrate of RIP3 and downstream effector of necrosome. RIP3-mediated phosphorylation of Pgam5 subsequently dephosphorylates and activates the mitochondrial fission regulator Drp1 on S637, leading to extensive mitochondrial fission, ROS production and subsequent necroptosis.^68^ It was notable that these data were obtained in HeLa cells. However, recent evidence suggests that neither Pgam5 nor Drp1 is involved in necroptosis, and that mitochondria may in fact be dispensable for the execution of the necroptotic program. It was demonstrated that knocking down or silencing of Pgam5 or Drp1 had no effect on necroptosis in a variety of cell lines, including MEF, L929, SVEC and NIH-3T3 cells.^62, 69, 70^ Therefore, there is some reason to suspect that the execution of necroptosis may be cell-type-specific.

## Caspase 8-Mediated Cleavage Inhibits Necroptosis

**Figure 4:**
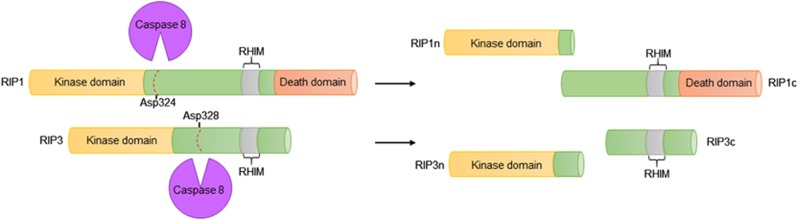
The caspase 8 cleavage sites on RIP1 and RIP3. Caspase 8 is a negative regulator of RIP1 and RIP3. It cleaves RIP1 on Asp324 and RIP3 on Asp328, which abolishes the activity of RIP1 and RIP3 and thus inhibits necroptosis

It has been reported that caspase 8 has opposing functions depending on its dimerization partners. On the one hand, caspase 8 can homodimerize with another caspase 8 to form a homodimer, wherein caspase 8 is fully processed and induces apoptosis. On the other hand, caspase 8 can heterodimerize with FLIP to form a heterodimer, wherein FLIP is primarily processed to induce cell survival.^71, 72^ In addition to the above-mentioned functions, recent studies have indicated that caspase 8 has a non-apoptotic role in regulating necroptosis. Caspase 8 has been shown to act as a negative regulator of necroptosis, as Casp8^−/−^RIP3^−/−^ double-knockout mice rescue the embryonic lethality of Casp8-deficient mice.^73^ Oberst *et al.*^74^ demonstrated that the caspase 8-FLIP heterodimer is engaged to inhibit necroptosis without triggering apoptosis. In the presence of FLIP, caspase 8 is unable to initiate apoptosis but maintains sufficient basal protease activity to cleave RIP1 and RIP3. These cleavage sites are identified to be Asp324 in RIP1 and Asp328 in RIP3 (Figure 4).^75, 76^ It is therefore possible that the caspase 8-FLIP heterodimer inhibits necroptosis by preventing stable RIP1–RIP3 association. However, another recent study identified CYLD as a key substrate cleaved by caspase 8 in the prevention of necroptosis.^32^ As cleavage of CYLD by caspase 8 does not require RIP1 or RIP3, it is suggested that this cleavage event occurs upstream of the necrosome assembly.

## Concluding Remarks

As the concept of programmed necrosis was first postulated in 1998, our view of this alternative cell death pathway has been broadened over the past two decades by identification of RIP1 and RIP3 downstream substrates, the initiators and inhibitors of necroptosis and physiological as well as pathological conditions involving necroptosis. As discussed in this review, the role of post-translational modification as an important regulatory mechanism of necroptosis has been established in many studies (Table 1).

Above all, ubiquitination is the most well-studied post-translational modification as a key regulator of necroptosis, and both RIP1 and RIP3 can be polyubiquitinated. K63-polyubiquitinated RIP1 is retained at the plasma membrane, which serves as a docking site to mediate NF-*κ*B activation and thus provides survival signal. On the contrary, K63-polyubiquitinated RIP3 supports the formation of the RIP1–RIP3 complex and subsequent induction of necroptosis. However, it remains to be seen which enzymes mediate the ubiquitination of RIP3. In addition to K63-linked chains, K11-linked ubiquitin chains mediated by cIAP1/2 and linear ubiquitin chains catalyzed by LUBAC also have an important function in pro-survival or pro-death pathways.

In the second place, the understanding of phosphorylation modification during necroptosis has been further explored with the current progress of related research. On account that both RIP1 and RIP3 are serine–threonine protein kinases, phosphorylation events are crucial for necrosome assembly and activity. Accordingly, the development of kinase inhibitors of necroptosis might provide a new therapeutic strategy for several human diseases associated with necroptosis. Although it was found that RIP1 and RIP3 could autophosphorylate themselves and also phosphorylate each other, many interesting questions remain to be answered regarding the exact phosphorylation sites. The next question worthy of discussion is whether the Pgam5–Drp1 axis is involved in necroptosis. As discussed above, it seems likely that this execution process of necroptosis is cell-type-specific.

In addition to the above-mentioned post-translational modifications, a previously unappreciated modification, GlcNAcylation, has recently attracted much attention. Li *et al.*^77^ revealed that the enteropathogenic *Escherichia coli* (EPEC) type III effector NleB can GlcNAcylate a conserved arginine in death domains of TNFR1, TRADD, FADD and RIP1 in EPEC-infected cells, which disrupts the assembly of the TNFR1 complex and blocks host cell apoptosis and necroptosis. This kind of unexpected and pathogen-related modification revealed that under certain circumstances, other types of post-translational modification may also be involved in the process of necroptosis. Currently, it is still controversial whether acetylation mediates necroptosis. With the deepening study of necroptosis, it is reasonable for us to believe that this problem will be solved in the near future.

## Figures and Tables

**Table 1:** The post-translational regulators of necroptosis

**Regulator**	**Category**	**Target**	**Function**	**Outcome**	**Reference**
A20	Znf domain: E3 ubiquitin ligase	RIP1	K48-linked polyubiquitination K63-linked polyubiquitination	Proteasomal degradation Inhibit cell death	^25^ ^26^
	OTU domain: deubiquitinating enzyme	RIP1 RIP3	Deubiquitination of K63-Ub chains Deubiquitination of K63-Ub chains	Promote cell death Inhibit necroptosis	^25^ ^50^
USP2a, USP4 and USP21	Deubiquitinating enzymes	RIP1	Deubiquitination of K63-Ub chains	Promote cell death	^27, 28, 29^
CYLD	Deubiquitinating protease	RIP1	Deubiquitination of RIP1	Promote necroptosis	^33, 34, 35^
cIAP1/2	E3 ubiquitin ligase	RIP1	K63, K48 and K11-linked polyubiquitination	Inhibit cell death	^36, 37, 38, 39^
TRAF2	E3 ubiquitin ligase	RIP1	K63-linked polyubiquitination	Inhibit cell death	^40, 41^
LUBAC	Linear ubiquitin ligase	RIP1?	Head-to-tail ubiquitination	Inhibit cell death	^47, 48, 49^
PKA, PKC or Jnk	Protein kinases	RIP1	Ser89 phosphorylation	Inhibit necroptosis	^55^
IKK*α*/IKK*β*	Protein kinases	RIP1	S25, S166, S296, S331 or S416 phosphorylation	Inhibit necroptosis	^56^
Ppm1b	Phosphatase	RIP3	Dephosphorylation of RIP3	Inhibit necroptosis	^59^
Caspase 8	Cysteine protease	CYLD, RIP1 and RIP3	Cleavage of CYLD at Asp215, RIP1 at Asp324 and RIP3 at Asp328	Inhibit necroptosis	^32, 73, 74, 75, 76^
NleB	GlcNAc transferase	TNFR1, TRADD, FADD and RIP1	GlcNAcylation death domains of TNFR1, TRADD, FADD and RIP1	Inhibit cell death	^77^

## Abbreviations

RIP1: receptor-interacting protein 1
RIP3: receptor-interacting protein 3
TNF: tumor necrosis factor
TLR: Toll-like receptor
MLKL: mixed lineage kinase domain like protein
Pgam5: phosphoglycerate mutase family member 5
NF-*κ*B: nuclear factor-*κ*B
DUB: deubiquitinating enzyme
Znf: Zinc-finger
USP: ubiquitin-specific protease
CYLD: cylindromatosis
TAK1: TGF-*β*-activated Kinase 1
TRAF2: TNF receptor-associated protein 2
cIAP: cellular inhibitor of apoptosis protein
LUBAC: linear ubiquitin chain assembly complex
HOIL1: heme-oxidized IRP2 ubiquitin ligase 1
HOIP: HOIL1-interacting protein
TNFR1: TNF receptor 1
TRADD: TNF receptor-associated death domain
FADD: Fas-associated death domain
PKA: protein kinase A
PKC: protein kinase C
Jnk: c-Jun N-terminal kinase
IKK: inhibitor of NF-*κ*B kinase
Ppm1b: protein phosphatase 1b
Drp1: dynamin-related protein 1
GlcNAcylation: N-acetylglucosaminylation
EPEC: enteropathogenic *Escherichia coli*
Nec-1: necrostatin-1
